# Preparation of SnIn_4_S_8_/TiO_2_ Nanotube Photoanode and Its Photocathodic Protection for Q235 Carbon Steel Under Visible Light

**DOI:** 10.1186/s11671-020-03447-1

**Published:** 2021-01-12

**Authors:** Hong Li, Weizhe Song, Xingqiang Cui, Yanhui Li, Baorong Hou, Lianjun Cheng, Pengfei Zhang

**Affiliations:** 1 State Key Laboratory of Bio-Fibers and Eco-Textiles, College of Mechanical and Electrical Engineering, Qingdao University, No. 308 Ningxia Road, Qingdao, 266071 People’s Republic of China; 2 Institute of Oceanology, Chinese Academy of Sciences, No. 7 Nanhai Road, Qingdao, 266071 People’s Republic of China; 3 Open Studio for Marine Corrosion and Protection, Pilot National Laboratory for Marine Science and Technology, No. 1 Wenhai Road, Qingdao, 266200 People’s Republic of China

**Keywords:** TiO_2_ nanotubes, SnIn_4_S_8_, Carbon steel, Photocathodic protection

## Abstract

TiO_2_ is an attractive semiconductor suitable for photocathodic protection, but its weak absorption of visible light and low quantum yield limit its usage. Here, a new heterostructured SnIn_4_S_8_ nanosheet/TiO_2_ nanotube photoanode was prepared and its photocathodic protection performance was analyzed. SnIn_4_S_8_ nanosheets were uniformly deposited on the surface of the TiO_2_ nanotube via a solvothermal treatment. The SnIn_4_S_8_**/**TiO_2_ composite exhibited better photocathodic protection performance compared with pure TiO_2_ nanotubes, owing to its good visible-light response and photogenerated carrier separation efficiency. Moreover, the composite exhibited a maximum photocurrent density of 100 μA cm^−2^ for a 6 h solvothermal reaction under visible light irradiation. The negative shift of the photoinduced potential of Q235 carbon steel connected to the composite could reach 0.45 V versus SCE. Therefore, the SnIn_4_S_8_/TiO_2_ composite can offer efficient photocathodic protection for Q235 carbon steel against corrosion in 3.5 wt% NaCl solution. This work provides a new approach for the development of high-efficient photoanode materials for the photocathodic protection of metals.

## Introduction

With the high-speed growth of industrial technology, metal corrosion has become a global problem [1, 2]. Metal corrosion not only shortens the service life of the equipment but also causes huge economic losses, even catastrophic safety accidents and environmental problems. Specifically, Q235 carbon steel (CS) is prone to severe corrosion in NaCl solution [3]. Photocathodic protection is an environmentally friendly and cost-saving technology with great application potential for metal anti-corrosion [4], which uses clean solar energy unlike conventional anti-corrosion technology. In addition, semiconductor photoanode materials are not consumed like traditional sacrificial anodes. This technology uses semiconductor materials (TiO_2_ [5], g-C_3_N_4_ [6], ZnO [7, 8], SrTiO_3_ [9]) to harvest solar photons and convert light energy into electricity to drive reduction chemical reactions efficiently, thereby effectively alleviating metal corrosion.

TiO_2_ has been widely used as a photoelectrode material for photocathodic protection due to its wide application range to catalyze various redox reactions, as well as its low cost, non-toxicity, and high chemical and photochemical stability [10–12]. However, individual TiO_2_ materials can only be induced by UV light as a result of their broad bandgap (3.0 eV for rutile, 3.2 eV for anatase). In addition, the available photoelectrons are reduced due to the rapid recombination of photogenerated carriers. To overcome the above disadvantages, numerous approaches have been proposed to heighten the photocathodic protection ability of TiO_2_-based photoelectrodes. These approaches include surface modification [13], designing highly ordered TiO_2_ nanotubes (NTs) [14], doping with metals or non-metals [15–18], and constructing heterojunctions [19–21]. More specifically, constructing heterojunctions by combining with other materials has proven to be a valid method for improving the photoelectrochemical properties of TiO_2_. The materials used for this strategy include metal oxides (In_2_O_3_ [22], MoO_3_ [23], Bi_2_O_3_ [24], WO_3_ [25, 26], RuO_2_ [27, 28]), metal sulfides and selenides (Ag_2_S [29], Bi_2_S_3_ [30], Ag_2_Se [31]), graphene [32–34], Co(OH)_2_ [35], and ZnFeAl-layered double hydroxides [36].

In addition, ternary and quaternary chalcogenides and selenium compounds, such as Cu_2_AgInS_4_ [37], Cu_2_AgInSe_4_ [38], and Cu_2_ZnSnSe_4_ [39], have received considerable attention in recent years due to their good photostability, strong absorption in the visible light range, and good electron transport properties. These materials have shown high photocatalytic activity to improve the photoconversion efficiency of quantum dot-sensitized solar cells. Stannum indium sulfide (SnIn_4_S_8_) is a ternary chalcogenide semiconductor [40] that shows promising applications in heavy metal reduction and the photocatalytic degradation of organics and pharmaceutical wastewater due to its good chemical stability and strong absorption of visible light [41, 42]. The physical and chemical ability of nanomaterials is mainly determined by their size, structure, and morphology. SnIn_4_S_8_ nanosheets have a large specific surface area, which may facilitate visible-light absorption [43] and reduce film resistance due to the fast charge transfer between the nanosheet and the electrolyte [44]. In addition, SnIn_4_S_8_ nanosheets have a relatively negative conduction band, which is beneficial to providing photocathodic protection for metals with a negative self-corrosion potential. Therefore, SnIn_4_S_8_ nanosheet-modified TiO_2_ NTs may show improved photoelectrochemical and photocathodic protection performance. However, there are only a few reports on the fabrication and cathodic protection application of SnIn_4_S_8_ nanosheet/TiO_2_ nanotube composites.

In the present study, a SnIn_4_S_8_ nanosheet/TiO_2_ NT heterojunction film was synthesized through a solvothermal reaction and subsequent electrochemical anodic oxidation method. The photocathodic protection performance and mechanism of SnIn_4_S_8_/TiO_2_ composites for Q235 CS were systematically studied.

## Methods

### Fabrication of SnIn_4_S_8_/TiO_2_ Photoelectrodes

TiO_2_ NTs were prepared on a titanium sheet (1 cm × 4 cm) by an electrochemical anodic oxidation process. The titanium sheet was cleaned after chemical polishing and anodized under 30 V for 30 min using ethylene glycol and 0.45 wt% NH_4_F and 8 wt% H_2_O as an electrolyte solution. A platinum sheet was used as the cathode. The sample was rinsed with deionized water and dried at 60 °C. TiO_2_ NTs were then acquired after heat treatment under 450 °C for 1.5 h.

**Fig. 1:**
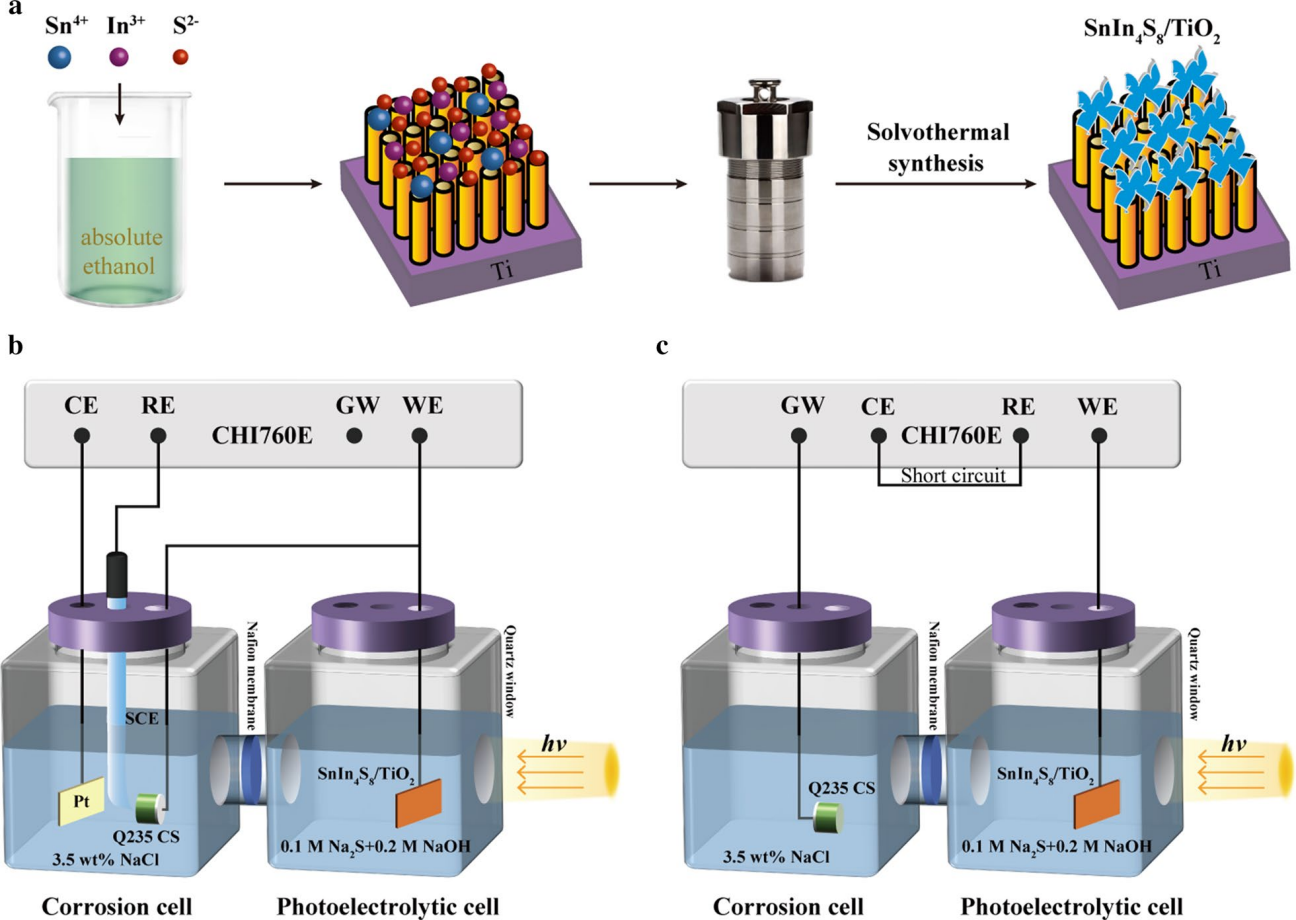
**a** Schematic illustration of the synthetic process for the preparation of the SnIn_4_S_8_/TiO_2_ composite film; schematic illustration of the experimental devices for photoelectrochemical measurements including **b** OCP variations and **c** photocurrent density variations

SnIn_4_S_8_/TiO_2_ NTs photoelectrodes were fabricated by depositing SnIn_4_S_8_ nanosheets on the surface of the TiO_2_ NTs through a simple solvothermal process (Fig. 1a). Typically, 0.05 mmol SnCl_4_·5H_2_O (0.0175 g), 0.2 mmol InCl_3_ (0.0587 g), and 0.5 mmol thioacetamide (0.0375 g) were added to 80 mL of absolute ethanol and stirred until the solution was homogeneous. The above solution and titanium sheet with fabricated TiO_2_ NTs were placed on the bottom of a 100 mL Teflon-lined stainless steel autoclave at 180 °C for 3–12 h. Then, the sample was cleaned repeatedly with absolute ethanol and dried at 70 °C for 4 h. The synthesized composites were marked as 3 h SnIn_4_S_8_/TiO_2_, 6 h SnIn_4_S_8_/TiO_2_, 9 h SnIn_4_S_8_/TiO_2_, 12 h SnIn_4_S_8_/TiO_2_, respectively. For comparison, SnIn_4_S_8_ nanosheets were prepared on the titanium sheet using the same procedure.

### Characterization

The morphology, microstructure, and elemental composition of the fabricated photoelectrodes were studied using a Hitachi SU8220 scanning electron microscope (SEM), high-resolution transmission electron microscope (HRTEM, JEOL JEM-2010), and energy dispersive spectrometer (EDS), respectively. The crystal structures of the prepared photoelectrodes were evaluated by X-ray diffraction (XRD) (D8-advance, Bruker AXS Co.) with Cu Kα irradiation at a wavelength of 0.15406 nm. The surface composition and chemical states were evaluated by X-ray photoelectron spectroscopy (XPS, ESCALAB 250XI, Thermo Scientific Co.) using Al Kα radiation. The optical characteristics were analyzed using a UV–Vis diffuse reflectance spectrophotometer (Hitachi UH4150). The photoluminescence (PL) spectra of the prepared photoelectrodes were measured on an FLS980 Series fluorescence spectrometer. Fourier transform infrared (FTIR) analyses were conducted on a Varian Scimitar 1000 spectrophotometer.

### Photoelectrochemical Tests

Photoelectrochemical tests were performed at room temperature on an electrochemical workstation (CHI760E, Chenhua Instrument Shanghai Co., Ltd.). The visible light source was a 300 W xenon light (PLS-SXE 300C, Perfect light Beijing Co., Ltd.) with a 420 nm cut-off filter. A Q235 CS electrode acted as the metal sample for testing, which was made via embedding a Q235 CS sample (1 cm × 1 cm × 1 cm) in an epoxy resin. The composition is as follows: 0.18% C; 0.28% Si; 0.035% S; 0.04% P; 0.55% Mn, and 98.915% Fe.

Figure 1b shows the schematic illustration of the experimental device for the measurement of the variations of the open circuit potential (OCP) of the Q235 CS coupled to the prepared photoelectrodes. The device consists of a photoelectrolytic cell and a corrosion cell, which were connected by a Nafion membrane. The prepared photoelectrodes were placed in the photoelectrolytic cell containing 0.1 M Na_2_S and 0.2 M NaOH solution, and the Q235 CS was set in the corrosion cell. The Q235 CS linked to the photoelectrodes via an external Cu wire was employed as the working electrode, while a saturated calomel electrode (SCE) and a platinum sheet were utilized as the reference electrode and the contrast electrode, respectively. The photoelectrodes were shined by visible light through a quartz window. The Tafel curves were tested from − 0.25 and + 0.25 V relative to the OCP at a sweep rate of 0.5 mV s^−1^. The electrochemical impedance spectroscopy (EIS) spectra were tested in a frequency range from 10^5^ to 10^−2^ Hz. The amplitude of the AC signal was 10 mV. Figure 1c displays the schematic illustration of the experimental device for the photocurrent density variation under intermittent visible light illumination. The photoelectrodes served as the working electrode, and Q235 CS was linked to the ground by a Cu wire. The contrast and reference electrode were short-circuited by a thin wire.

## Results and Discussion

### Morphologies and Chemical Compositions

**Fig. 2:**
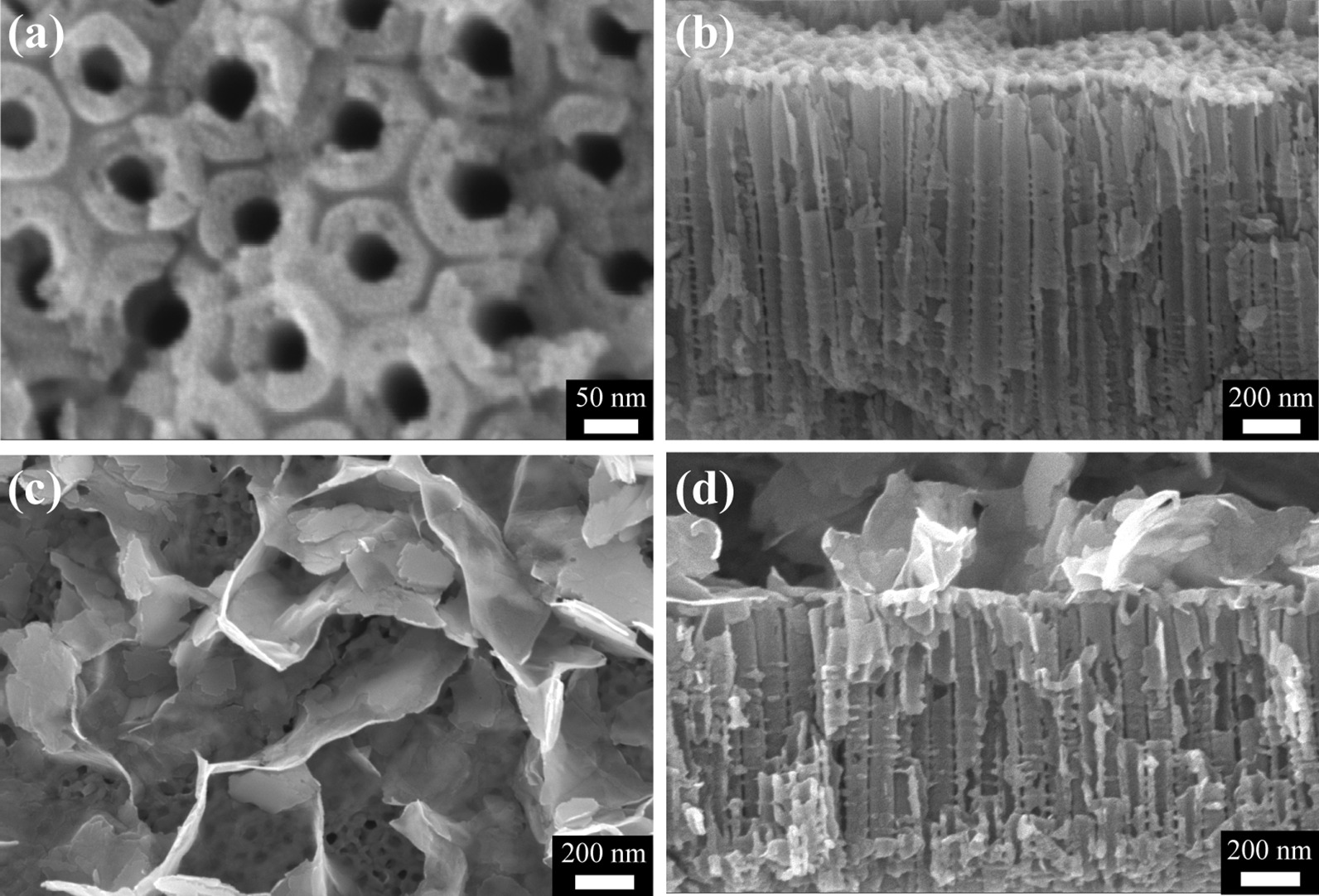
**a** Topographic and **b** cross-sectional SEM images of the TiO_2_ NT films, **c** topographic and **d** cross-sectional SEM images of the SnIn_4_S_8_/TiO_2_ composite film

Figure 2 reveals the morphologies of the TiO_2_ and SnIn_4_S_8_/TiO_2_ composite films. As shown in Fig. 2a, b, a well-aligned and highly ordered NTs film was successfully formed on the titanium surface through an electrochemical anodic oxidation process. The average inner diameter of TiO_2_ NTs was approximately 50 nm, the wall thickness was approximately 25 nm, and the tube length was approximately 1300 nm. It can be observed that numerous curved SnIn_4_S_8_ nanosheets were attached to the TiO_2_ surface after a solvothermal reaction for 6 h (Fig. 2c, d). The nanostructure composed of thin nanosheets and large hierarchical pores was conducive to the improvement of the light harvestability due to multiple light scattering, which may facilitate the charge transfer in the photochemical reaction and enhance the photocathodic protection performance of the SnIn_4_S_8_/TiO_2_ composite.

**Fig. 3:**
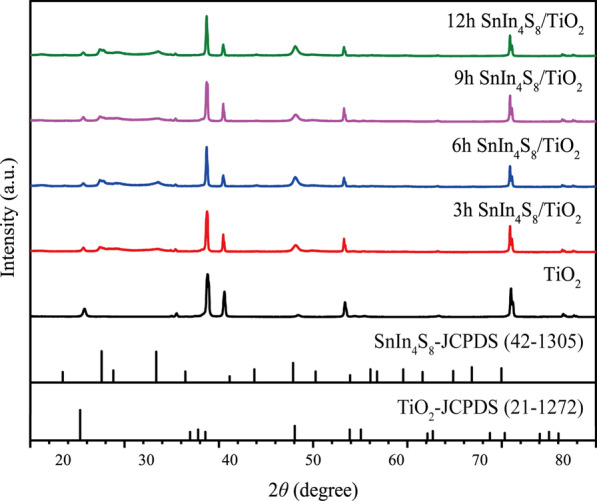
XRD spectra of pure TiO_2_ and the different SnIn_4_S_8_/TiO_2_ composites

Figure 3 shows the XRD spectra of the synthesized TiO_2_ and the SnIn_4_S_8_/TiO_2_ composite films. For pure TiO_2_, except for the characteristic peaks of the titanium substrate, the peaks at 25.3°, 48.1°, and 53.9° were indexed to the anatase (101), (200), and (105) lattice planes, respectively (JCPDS Card No. 21-1272). For the SnIn_4_S_8_/TiO_2_ composites, the XRD patterns of the obtained samples were similar. Compared with pure TiO_2_, three other diffraction peaks were observed in the XRD spectra of the SnIn_4_S_8_/TiO_2_ composites. The peaks at 27.5°, 28.4°, and 33.0° were assigned to the lattice planes (311), (222), and (400) of tetragonal SnIn_4_S_8_, respectively (JCPDS Card No. 42-1305). No peaks corresponding to binary sulfides and oxides were detected, confirming the high purity of the SnIn_4_S_8_/TiO_2_ composites.

**Fig. 4:**
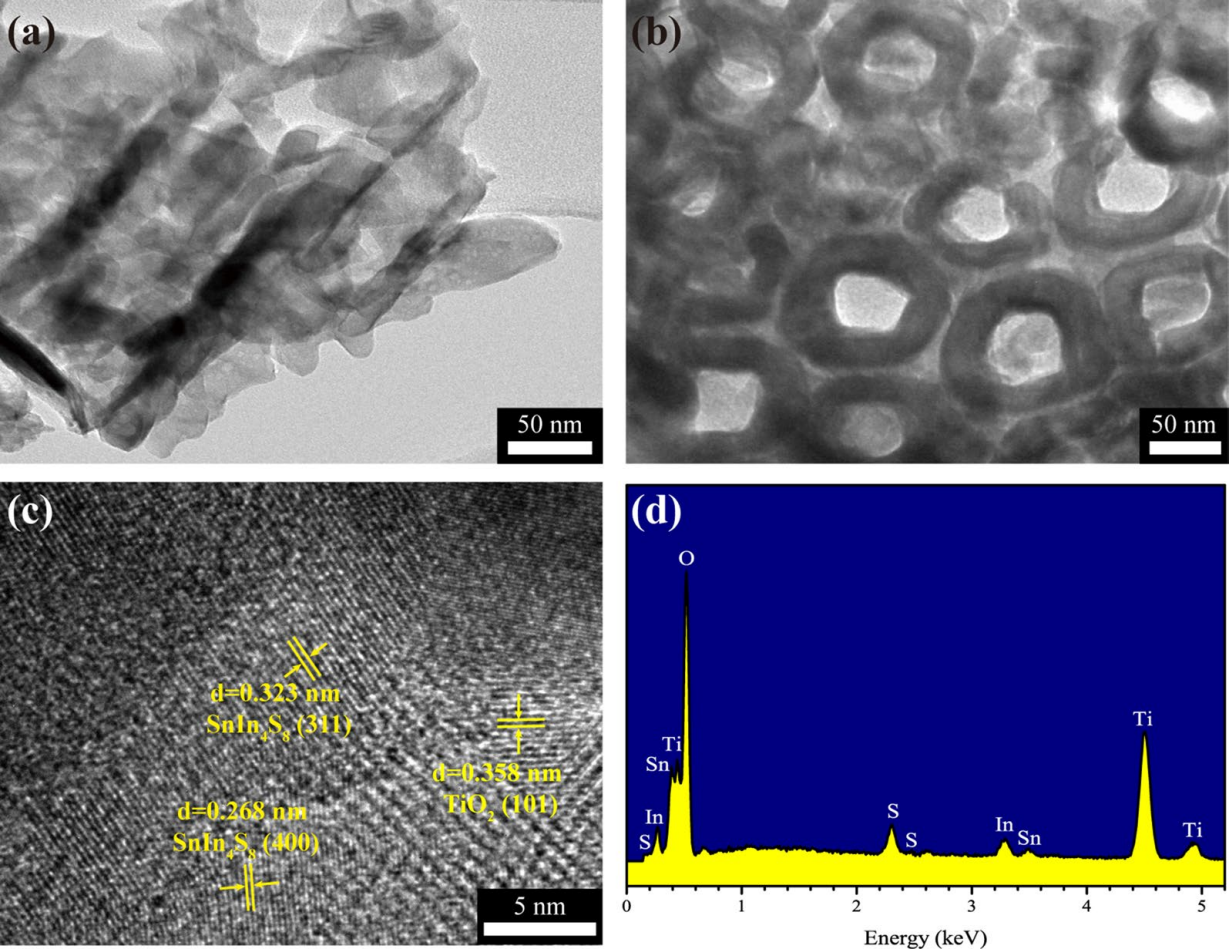
**a**, **b** TEM, and **c** HRTEM images, and **d** EDS spectrum of the SnIn_4_S_8_/TiO_2_ composite film

To further study the microstructures of the SnIn_4_S_8_/TiO_2_ composite, TEM images were analyzed as shown in Fig. 4a, b, confirming that the SnIn_4_S_8_ nanosheets were attached to the TiO_2_ NTs surface. The wall thickness and inner diameter of the TiO_2_ NTs were approximately 25 and 50 nm, respectively, which was consistent with the SEM results. Figure 4c is an HRTEM image taken at the edge of the surface of the composite in Fig. 4a, which displays a close contact between the TiO_2_ NTs and the SnIn_4_S_8_ nanosheets. The lattice fringe with a spacing of 0.358 nm was assigned to the anatase (101) plane of TiO_2_ (JCPDS Card No. 21-1272). The lattice fringe with a spacing of 0.268 nm and 0.323 nm was indexed to the tetragonal (400) and (311) planes of SnIn_4_S_8_ (JCPDS Card No. 42-1305), respectively. The results confirmed that SnIn_4_S_8_ nanosheets were constructed on the TiO_2_ NTs surface by the solvothermal process. The corresponding EDS spectrum (Fig. 4d) demonstrated that the SnIn_4_S_8_/TiO_2_ composite film was composed of Ti, O, Sn, S, and In elements.

**Fig. 5:**
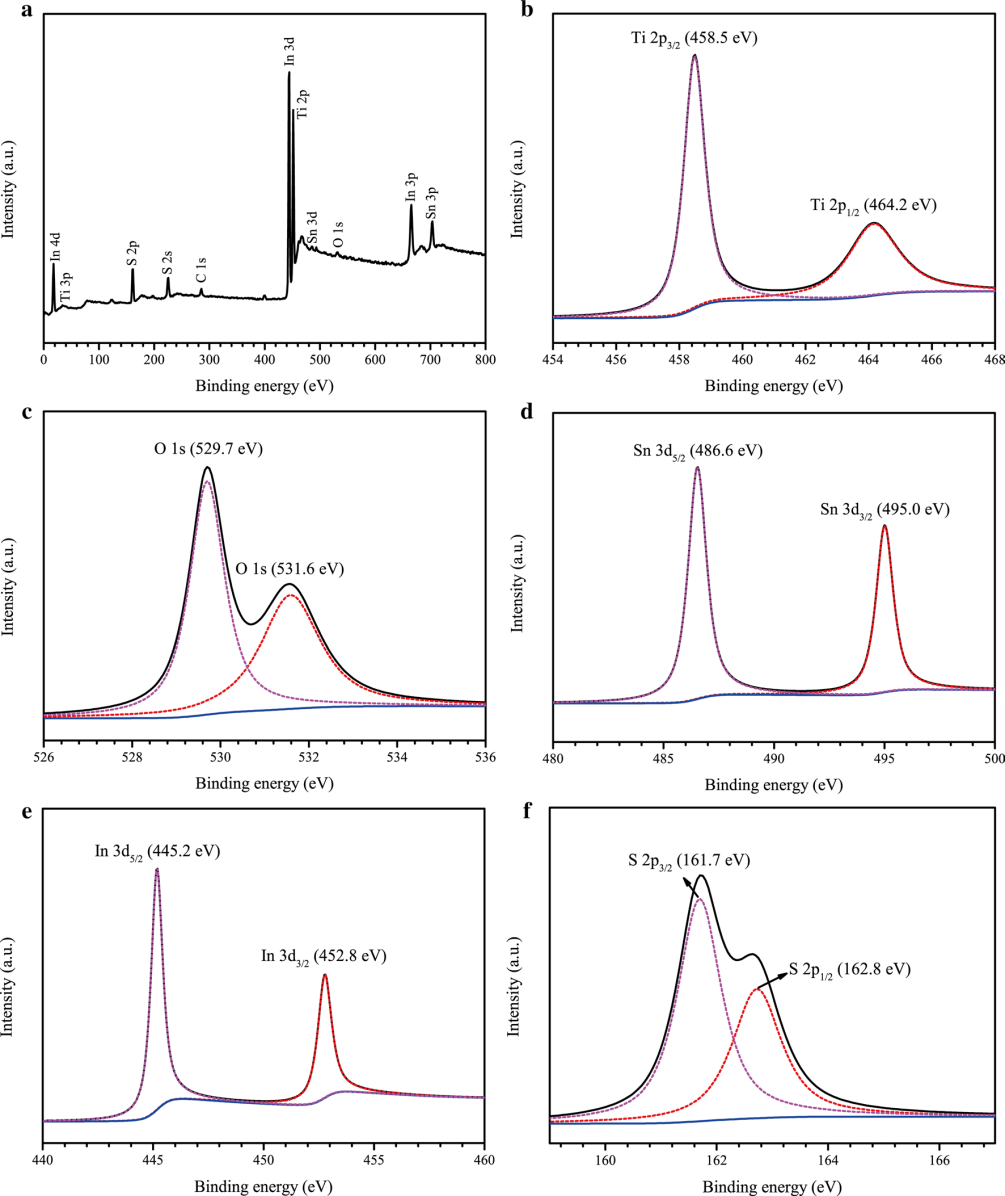
**a** XPS survey spectrum of the SnIn_4_S_8_/TiO_2_ composite film; high-resolution XPS spectra of **b** Ti 2*p*, **c** O 1*s*, **d** Sn 3*d*, **e** In 3*d* and **f** S 2*p*

The surface components and chemical states of SnIn_4_S_8_/TiO_2_ were studied by XPS. From the XPS survey spectrum (Fig. 5a), it was confirmed that Ti, O, Sn, In, S elements were present in the composite, which was consistent with the EDS result. Moreover, a characteristic peak corresponding to the C element was observed, which was caused by exposure to atmospheric pollution. Figure 5b displays the high-resolution XPS spectrum of Ti 2*p*. The peaks of the binding energy at 458.5 and 464.2 eV were coincident with Ti 2*p*_3/2_ and Ti 2*p*_1/2_, respectively, which are characteristic peaks of Ti^4+^ [45]. This indicated that Ti^4+^ existed in the SnIn_4_S_8_/TiO_2_ composite. Figure 5c shows the peaks at 529.7 and 531.6 eV, which correspond to lattice oxygen and adsorbed oxygen, respectively [46]. The lattice oxygen represented the Ti–O, indicating the existence of TiO_2_ in the composite. The adsorbed oxygen came from H_2_O adsorbed on the surface of the composite, suggesting that the surface of the composite was rich in oxygen vacancies. Figure 5d reveals that the peak centered at 495.0 and 486.6 eV belonged to Sn 3*d*_3/2_ and Sn 3*d*_5/2_, respectively [40], indicating that the valence state of Sn was + 4. As shown in Fig. 5e, the binding energies of 445.2 and 452.8 eV were assigned to In 3*d*_5/2_ and In 3*d*_3/2_, respectively, suggesting the existence of In^3+^ in the composite [47]. The peaks at 162.8 and 161.7 eV in Fig. 5f correspond to S 2*p*_1/2_ and S 2*p*_3/2_, respectively, showing that S element existed mainly in the form of S^2−^ in the SnIn_4_S_8_/TiO_2_ NTs [48]. The above results further indicate that a SnIn_4_S_8_/TiO_2_ NTs heterojunction film was successfully synthesized by the combination of a solvothermal and electrochemical anodic oxidation process, which was in good agreement with the results from SEM, TEM, and XRD analyzes.

To investigate the growth process of the SnIn_4_S_8_ nanosheets, the SEM images of the composites fabricated by the solvothermal process for 3, 9, and 12 h were observed. When the reaction time was 3 h, small SnIn_4_S_8_ nanosheets appeared on the surface of the TiO_2_ NTs in the obtained composite (Additional file 1: Fig. S1a, d). After the time reached 6 h, the size and thickness of the nanosheets increased (Fig. 2c, d). However, once the solvothermal reaction time exceeded 9 h, the SnIn_4_S_8_ nanosheets completely covered the TiO_2_ surface and blocked the mouth of the TiO_2_ NTs, which was harmful to the photogenerated charge separation (Additional file 1: Fig. S1b, c, e, f). The main reactions during the fabrication process are as follows:

1 $${\text{CH}}_{{3}} {\text{CSNH}}_{{2}} + {\text{H}}_{2} {\text{O}} \to {\text{CH}}_{{3}} {\text{CONH}}_{{2}} { + \text{ H}}_{2} {\text{S}}$$

2 $${\text{H}}_{2} {\text{S}} \to {\text{2H}}^{ + } { + \text{ S}}^{{2-}}$$

3 $${\text{Sn}}^{{4 + }} { + \text{ 4In}}^{{3 + }} { + \text{ 8S}}^{{2-}} \to {\text{SnIn}}_{{4}} {\text{S}}_{{8}}$$

4 $${\text{SnIn}}_{{4}} {\text{S}}_{{8}} { + \text{ TiO}}_{{2}} \to {\text{SnIn}}_{{4}} {\text{S}}_{{8}} {\text{/TiO}}_{{2}}$$

### Optical Characteristics

**Fig. 6:**
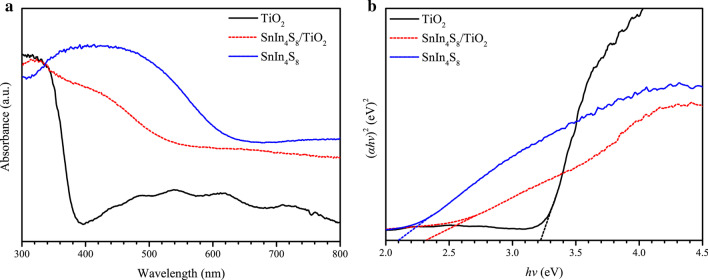
UV–visible diffuse reflectance spectra of TiO_2_, SnIn_4_S_8_, and SnIn_4_S_8_/TiO_2_ composite film

The optical characteristics of the fabricated photoelectrodes were analyzed by UV–visible diffuse reflectance spectra (Fig. 6a). The light absorption of pure TiO_2_ was mainly in the UV region, and the absorption edge was approximately 385 nm, which was attributed to the inherent absorption of TiO_2_. Moreover, the light absorption of TiO_2_ in the visible region may be ascribed to light scattering caused by cracks or holes in the NTs [49]. The absorption edge of SnIn_4_S_8_ and the SnIn_4_S_8_/TiO_2_ composite appeared near 590 and 535 nm, respectively. The bandgap (*E*_g_) values of the photoelectrodes were computed using the following equation [50]:

5 $$(\alpha {\text{h}}v)^{2} = {\text{A}}({\text{h}}v - E_{{\text{g}}} )$$

where *α*, *hv*, and *A* imply the absorption coefficient, photon energy, and characteristic constant, respectively. The plot of (*αhv*)^2^ versus *hv* for calculating the bandgap is displayed in Fig. 6b. The *E*_g_ value of pure TiO_2_ was 3.22 eV, which was similar to that of anatase TiO_2_ (3.2 eV) [51]. The *E*_g_ value of SnIn_4_S_8_ and the SnIn_4_S_8_/TiO_2_ composites was evaluated to be 2.1 and 2.32 eV, respectively. These results indicated that the absorption capacity of the SnIn_4_S_8_/TiO_2_ composite was enhanced in the visible light region, which is beneficial to the utilization of solar energy and improvement of the photocathodic protection performance.

The PL spectra measurement was used to examine the separation, transfer, and recombination of photogenerated carriers of semiconductor materials [52]. The weaker PL intensity means the lower the recombination rate of photogenerated carriers [53]. The PL intensity of the SnIn_4_S_8_/TiO_2_ composite was lower than that of TiO_2_ NTs (Additional file 1: Fig. S2), indicating that the sensitization of the SnIn_4_S_8_ nanosheets could effectively restrain the recombination of photogenerated carriers in the TiO_2_ NTs.

### Photoelectrochemical Performance

**Fig. 7:**
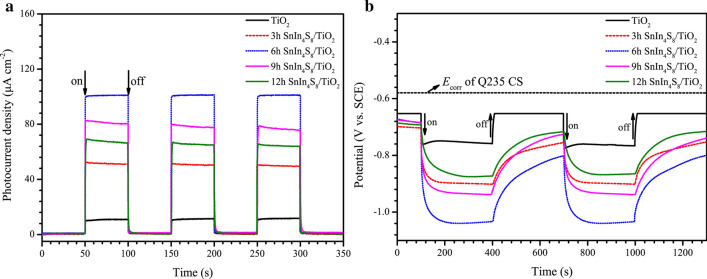
Variations in the **a** photocurrent density and **b** potential of the Q235 CS electrode coupled to the prepared TiO_2_ and different SnIn_4_S_8_/TiO_2_ photoanodes under intermittent visible light irradiation

Photocurrent density is a significant parameter used to investigate the photocathodic protection properties of semiconductor photoanodes. The greater the photocurrent density of the metal coupled to the photoanode, the better the photoelectric conversion performance and the photocathodic protection effect of the photoanode [54]. Figure 7a displays the photocurrent density changes of a Q235 CS electrode coupled to pure TiO_2_ or SnIn_4_S_8_/TiO_2_ NTs photoanodes. Before the lamp was turned on, the photocurrent densities of the metal electrode coupled to the photoanodes were almost zero, indicating no photogenerated electrons were transferred to the steel electrode at this time. When the light was turned on, both pure TiO_2_ and the SnIn_4_S_8_/TiO_2_ composite photoanodes exhibited a photocathodic protection current for the Q235 CS electrode. This was because photogenerated electrons were carried from the photoanodes to the surface of the steel electrode. The SnIn_4_S_8_/TiO_2_ composite fabricated by the 6 h solvothermal reaction demonstrated the greatest photocurrent density (about 100 μA cm^−2^), which was approximately 8 times larger than pure TiO_2_. This indicates that the modification of the SnIn_4_S_8_ nanosheets remarkably enhanced the photoelectron separation and transmission efficiency of the TiO_2_ NTs. The photocurrent densities decreased in the order of 6 h SnIn_4_S_8_/TiO_2_ > 9 h SnIn_4_S_8_/TiO_2_ > 12 h SnIn_4_S_8_/TiO_2_ > 3 h SnIn_4_S_8_/TiO_2_ > TiO_2_. As the time of the solvothermal reaction increased, the SnIn_4_S_8_ nanosheets absorbed more light energy to generate sufficient photoelectrons, thus exhibiting a larger photocurrent. However, when the solvothermal reaction time exceeded 6 h, the increase in the thickness of the nanosheets increased the transfer distance of the photoelectrons in the nanosheets [55, 56]. In addition, the TiO_2_ NTs cannot absorb light to generate photoelectrons since the oversized SnIn_4_S_8_ nanosheets blocked their mouth. This ultimately led to a decrease in photoelectrons transferred to the Ti substrate, which decreased the photocurrent of the composite.

The variation of the potential of the metal coupled to the semiconductor photoanode is another considerable parameter used to investigate the photocathodic protection property of the photoanodes [57]. Photo-excitation of the photoanodes generates electrons, which are transferred to the surface of the steel and reduce its potential, and then protect the steel electrode. Figure 7b demonstrates the OCP changes of Q235 CS linked to TiO_2_ and SnIn_4_S_8_/TiO_2_ NTs photoanodes. The potentials of the coupled Q235 CS decreased slightly before illumination due to the galvanic effect. After the light was turned on, the photogenerated potentials of the Q235 CS electrode coupled to TiO_2_ and SnIn_4_S_8_/TiO_2_ NTs had undergone significant negative shifts. The order of the potential drop was 6 h SnIn_4_S_8_/TiO_2_ (0.45 V vs. SCE) > 9 h SnIn_4_S_8_/TiO_2_ (0.36 V vs. SCE) > 3 h SnIn_4_S_8_/TiO_2_ (0.32 V vs. SCE) > 12 h SnIn_4_S_8_/TiO_2_ (0.30 V vs. SCE) > TiO_2_ (0.18 V vs. SCE). The potentials of Q235 CS linked to the SnIn_4_S_8_/TiO_2_ composites slowly increased after the light was turned off, and the coupled potentials were still much lower than that of bare Q235 CS. This indicated that the composites could continue to protect the steel for some time even in the absence of light due to the slow release of electrons stored in the composites. Therefore, combined with the results of photocurrent densities, it can be concluded that the SnIn_4_S_8_/TiO_2_ composites had better photocathodic protection for Q235 CS than pure TiO_2_. The optimal photocathodic protection was achieved for the SnIn_4_S_8_/TiO_2_ composite prepared for 6 h. In addition, the 6 h SnIn_4_S_8_/TiO_2_ composite photoanode displayed higher photocathodic protection properties than most photoanodes previously reported (Additional file 1: Table S1). The SnIn_4_S_8_/TiO_2_ composites mentioned in the following sections were obtained for the sample developed by 6 h of reaction time.

**Fig. 8:**
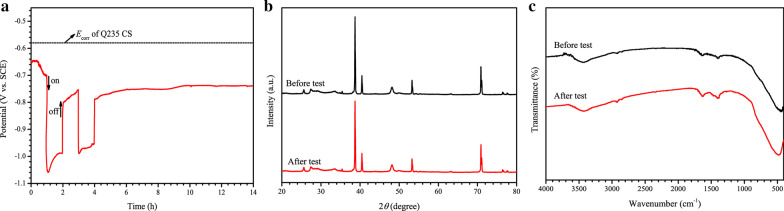
**a** Long-term potential changes of Q235 CS connected to the SnIn_4_S_8_/TiO_2_ composite under intermittent visible light illumination; **b** XRD patterns of the SnIn_4_S_8_/TiO_2_ composite before and after photoelectrochemical tests; **c** FTIR spectra of the SnIn_4_S_8_/TiO_2_ composite before and after photoelectrochemical tests

In order to investigate the stability of the SnIn_4_S_8_/TiO_2_ composite, the long term potential variation of a Q235 CS electrode connected to the photoanode under intermittent visible light illumination was analyzed. As displayed in Fig. 8a, the self-corrosion potential of the bare Q235 CS was − 0.58 V versus SCE in 3.5 wt % NaCl solution. When the CS was connected to the SnIn_4_S_8_/TiO_2_ composite, the potential dropped rapidly with visible light irradiation, which may be due to the transfer of photogenerated electrons from the composite to the steel. Under long term visible light irradiation, the potential of steel was stable at − 0.96 V versus SCE. This was only 0.07 V higher than the photogenerated potential obtained from the OCP results, indicating that the SnIn_4_S_8_/TiO_2_ composite had good stability. After the light irradiation was stopped, the potential of Q235 CS increased back to − 0.74 V versus SCE, which was still far from the self-corrosion potential, demonstrating that the composite can provide the steel with continuous protection in the dark state. Moreover, as shown in Fig. 8b, c, the XRD pattern and FTIR spectrum of the SnIn_4_S_8_/TiO_2_ composite after the photoelectrochemical test were consistent with those before the test, respectively, indicating that the composite possesses good photoelectrochemical stability.

**Fig. 9:**
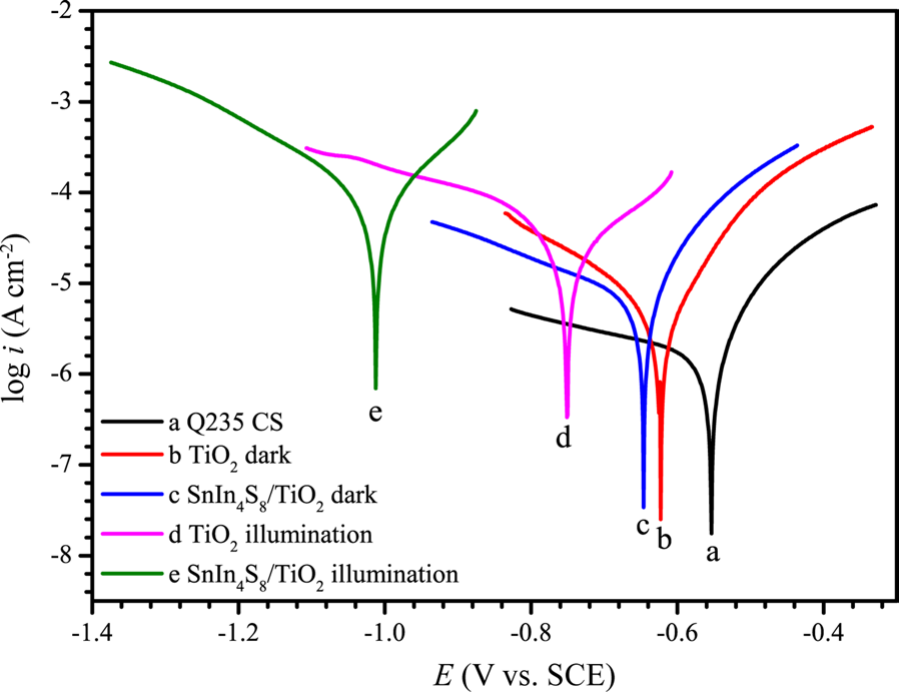
Tafel curves of Q235 CS in 3.5% NaCl solution under different conditions, **a** bare Q235 CS, **b** Q235 CS coupled to TiO_2_ NTs film in the dark, **c** Q235 CS coupled to SnIn_4_S_8_/TiO_2_ composite film in the dark, **d** Q235 CS coupled to TiO_2_ NTs film under visible light irradiation, and **e** Q235 CS coupled to SnIn_4_S_8_/TiO_2_ composite film under visible light irradiation

The Tafel curves of the pure Q235 CS, the Q235 CS electrode linked to the pure TiO_2_, and the 6 h SnIn_4_S_8_/TiO_2_ composite in dark conditions and under irradiation were tested to further evaluate the photoelectrochemical performance of the SnIn_4_S_8_/TiO_2_ composites (Fig. 9). Corrview software was utilized to compute the corrosion potential (*E*_corr_) and corrosion current (*i*_corr_), and the results are displayed in Table 1. Under visible light irradiation, after Q235 CS was linked to the pure TiO_2_ or the SnIn_4_S_8_/TiO_2_ composite electrode, the *E*_corr_ shifted negatively, indicating that photogenerated electrons effectively migrated from the photoelectrode to the steel electrode, thereby providing a photocathodic protection effect for the Q235 CS electrode. The *E*_corr_ of Q235 CS linked to the SnIn_4_S_8_/TiO_2_ composite (− 0.92 V vs. SCE) under irradiation was much lower than that of pure TiO_2_ (− 0.75 V vs. SCE), showing that the composite was able to provide Q235 CS with better cathodic protection than pure TiO_2_. The *i*_corr_ of Q235 CS linked to the SnIn_4_S_8_/TiO_2_ composite notably increased compared with that of bare Q235 CS under light irradiation (Table 1). This is as a result of the polarization of photoinduced electrons accelerating the rate of chemical reactions at the interface [58, 59].

**Table 1:** Electrochemical parameters obtained by the Tafel curves of Q235 CS, TiO_2_ in the dark, SnIn_4_S_8_/TiO_2_ in the dark, TiO_2_ under illumination, and SnIn_4_S_8_/TiO_2_ under illumination

Samples	*E*_corr_ (V vs. SCE)	*i*_corr_ (μA cm^−2^)
Q235 CS	− 0.554	1.658
TiO_2_^dark^	− 0.623	3.144
SnIn_4_S_8_/TiO_2_^dark^	− 0.646	5.313
TiO_2_^illumination^	− 0.751	18.94
SnIn_4_S_8_/TiO_2_^illumination^	− 1.011	63.10

**Fig. 10:**
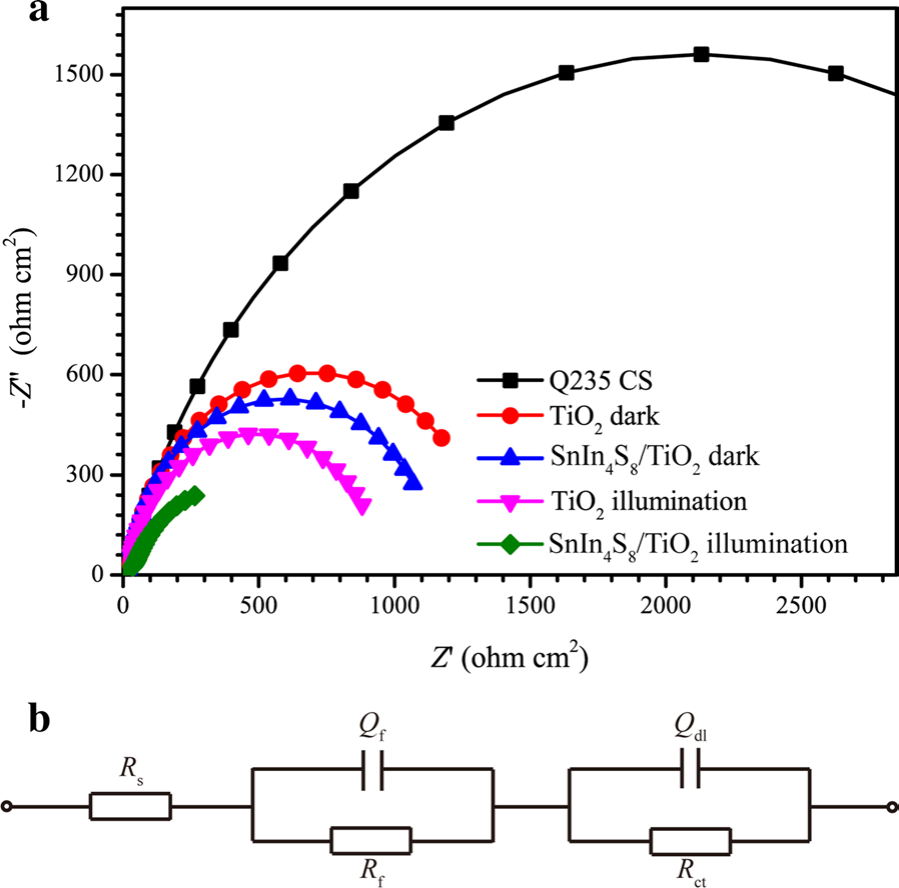
**a** Nyquist plots of bare Q235 CS, Q235 CS coupled to TiO_2_ NTs film, and SnIn_4_S_8_/TiO_2_ composite film under and after visible light irradiation, and **b** the corresponding equivalent circuit

EIS was used to further study the photogenerated carrier separation and transfer process of the SnIn_4_S_8_/TiO_2_ composite film and the corrosion resistance of the Q235 CS electrode. Figure 10a shows the Nyquist plots of Q235 CS, Q235 CS coupled to a TiO_2_ NT film, and the SnIn_4_S_8_/TiO_2_ composite film under and after visible light irradiation. The fitting circuit for EIS using ZSimpWin software consisted of an *R*_s_(*Q*_f_*R*_f_)(*Q*_dl_*R*_ct_) model, as demonstrated in Fig. 10b, where *R*_s_ indicates the electrolyte resistance, *Q*_f_ and *R*_f_ indicate the capacitance and resistance of the semiconductor film electrode at high frequencies, respectively, and *Q*_dl_ and *R*_ct_ indicate the electric double-layer capacitance and the charge transfer resistance at low frequencies, respectively.

The fitting impedance parameters of EIS using the equivalent circuit are also shown in Table 2. The diameter of the impedance arc of the coupled Q235 CS electrode under visible light was smaller than that of pure Q235 CS, and the *R*_ct_ values of the coupled Q235 CS electrode decreased significantly. The results indicate that the rate of the electrochemical reaction at the interface between the Q235 CS and the solution increased remarkably, which may be due to the migration of photoelectrons from the photoanode to the Q235 CS [60]. In addition, the *R*_ct_ value of the SnIn_4_S_8_/TiO_2_ composite was smaller than that of TiO_2_, which may be because the heterojunction formed by TiO_2_ and SnIn_4_S_8_ was conducive to the separation and migration of photoinduced charges. These results were consistent with those obtained from the Tafel curves (Fig. 9). Furthermore, the diameter of the impedance arc of the SnIn_4_S_8_/TiO_2_ composite under visible light was distinctly smaller than the impedance after the light was turned off, suggesting that more photoelectrons migrated from the SnIn_4_S_8_/TiO_2_ composite to Q235 CS under visible light irradiation. This demonstrates that the SnIn_4_S_8_/TiO_2_ composite could offer Q235 CS effective photocathodic protection in the presence of visible light.

**Table 2:** Fitting impedance parameters of Nyquist plots using the equivalent circuit in Fig. 10b

Samples	*R*_s_ (Ω cm^2^)	*Q*_f_		*R*_f_ (Ω cm^2^)	*Q*_dl_		*R*_ct_ (kΩ cm^2^)
		10^3^*Y*_01_(S^*n*^·Ω^−1^ cm^−2^)	*n*_1_		10^3^*Y*_02_(S^*n*^·Ω^−1^ cm^−2^)	*n*_2_	
Q235 CS	5.875	1.918	0.825	6.617	1.185	0.809	4.238
TiO_2_^dark^	5.220	1.603	0.813	20.84	3.080	0.934	1.343
SnIn_4_S_8_/TiO_2_^dark^	5.182	3.235	0.885	19.31	3.042	0.936	1.141
TiO_2_^illumination^	4.993	2.257	0.922	18.55	1.882	0.949	0.932
SnIn_4_S_8_/TiO_2_^illumination^	5.343	6.283	0.873	36.47	1.947	0.825	0.278

### Mechanism

**Fig. 11:**
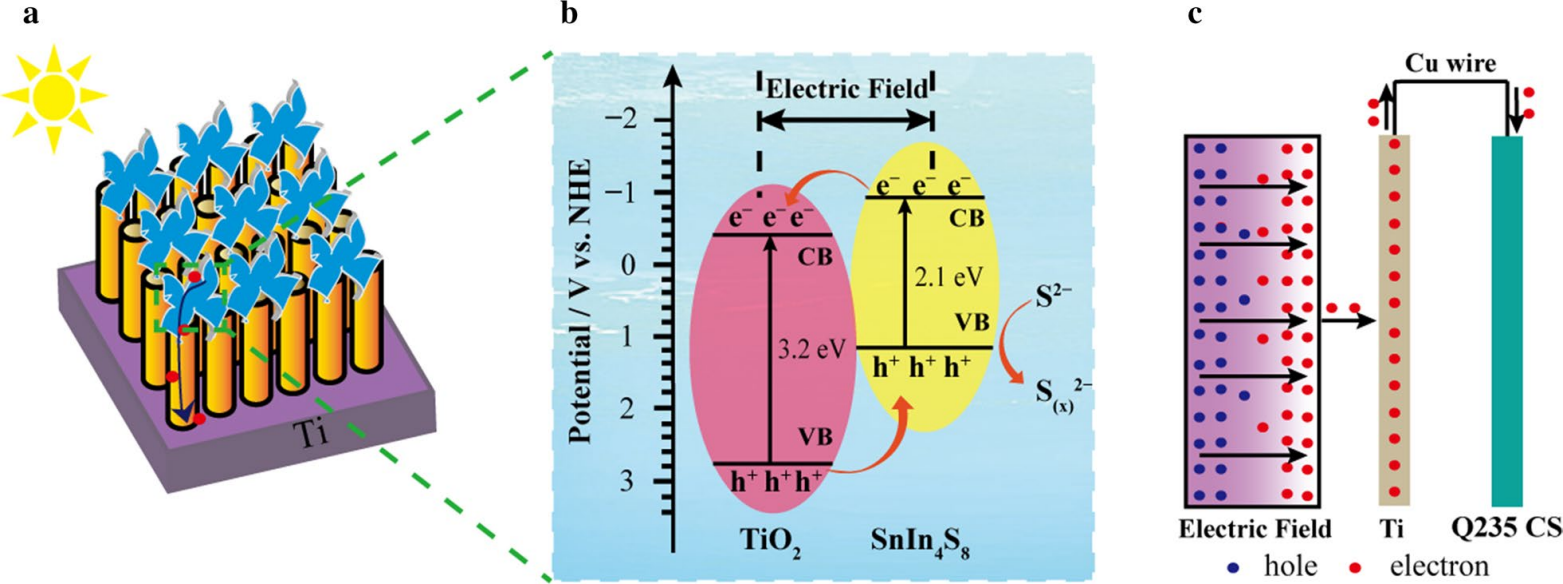
Proposed mechanisms for the enhanced photocathodic protection performance of the SnIn_4_S_8_/TiO_2_ composite for Q235 CS under visible light illumination

Figure 11 displays the proposed mechanism for the above-mentioned photocathodic protection of the SnIn_4_S_8_/TiO_2_ composites and the photoelectron separation and transfer process. When the visible light illuminated the SnIn_4_S_8_/TiO_2_ heterojunction, both the SnIn_4_S_8_ and TiO_2_ were excited to generate photoelectrons and holes. The photoelectrons migrated from the conduction band of SnIn_4_S_8_ to the conduction band of TiO_2_ because the potential of the former is much lower than that of TiO_2_ (Fig. 11a, b). Subsequently, these electrons migrated to the surface of the steel electrode driven by the electric field force because the potential was lower than its self-corrosion potential (Fig. 11c). This process inhibited the corrosion of the Q235 CS as a result of cathodic polarization. Meanwhile, the photogenerated holes moved from the valence band of TiO_2_ to the valence band of SnIn_4_S_8_. After the light was turned off, the stored electrons in the SnIn_4_S_8_/TiO_2_ composites migrated continually to the Q235 CS electrode, which realized the protection of steel in the dark state. In addition, the photogenerated holes reacted with S^2−^ in the electrolyte solution to form polysulfides (S_(x)_^2−^) [61], which could accelerate the photogenerated carrier separation.

## Conclusions

With the increasingly prominent energy crisis and environmental pollution caused by metal corrosion, there is an urgent need to develop highly efficient visible-light-responsive semiconductor photoanodes. In this study, a SnIn_4_S_8_**/**TiO_2_ nanotube photoanode was successfully fabricated via a solvothermal treatment and subsequent electrochemical anodic oxidation method. The results indicated that the nanostructure of the SnIn_4_S_8_**/**TiO_2_ composite consisted of thin nanosheets and large hierarchical pores, which were conducive to photogenerated carrier separation. The optical characteristic analysis showed that the absorption capacity of the SnIn_4_S_8_/TiO_2_ composite was enhanced in the visible light region. The composite fabricated by a solvothermal reaction for 6 h exhibited the optimal photocathodic protection performance. The photocurrent density of Q235 CS coupled with the 6 h SnIn_4_S_8_/TiO_2_ composite achieved 100 μA cm^−2^, which was approximately 8 times larger than pure TiO_2_. The maximum negative shift value of the photoinduced potential of Q235 CS could reach 0.45 V versus SCE. The excellent photocathodic protection effect of the SnIn_4_S_8_/TiO_2_ composite for Q235 CS suggests that the composite is a promising photoelectrode material for the photocathodic protection of metals.

## Supplementary information

**Additional file 1** **Fig. S1**. SEM images of the SnIn_4_S_8_ nanosheet/TiO_2_ composite films synthesized at 180 °C for (a), (d) 3 h, (b), (e) 9 h and (c), (f) 12 h. **Fig. S2.** PL spectra of TiO_2_ NTs and SnIn_4_S_8_/TiO_2_ composite. **Table S1.** Comparison of previously reported catalysts for photocathodic protection.

## Data Availability

All datasets are presented in the main paper.

## Abbreviations

CS: Carbon steel
NTs: Nanotubes
SnIn_4_S_8_: Stannum indium sulfide
SEM: Scanning electron microscope
HRTEM: High-resolution transmission electron microscope
EDS: Energy dispersive spectrometer
XRD: X-ray diffraction
XPS: X-ray photoelectron spectroscopy
PL: Photoluminescence
FTIR: Fourier transform infrared
OCP: Open circuit potential
SCE: Saturated calomel electrode
EIS: Electrochemical impedance spectroscopy
*E*_g_: Bandgap
*E*_corr_: Corrosion potential
*i*_corr_: Corrosion current
