# Supplementary material for: Preparation of SnIn4S8/TiO2 Nanotube Photoanode and Its Photocathodic Protection for Q235 Carbon Steel Under Visible Light
Source: Nanoscale Res Lett. 2021 Jan 12;16:10. doi: 10.1186/s11671-020-03447-1 (PMC7803867; doi:10.1186/s11671-020-03447-1)
Supplement: Supplementary file 1 — Additional file 1 Fig. S1. SEM images of the SnIn4S8 nanosheet/TiO2 composite films synthesized at 180 °C for (a), (d) 3 h, (b), (e) 9 h and (c), (f) 12 h. Fig. S2. PL spectra of TiO2 NTs and SnIn4S8/TiO2 composite. Table S1. Comparison of previously reported catalysts for photocathodic protection. [file 11671_2020_3447_MOESM1_ESM.docx]

**Preparation of SnIn_4_S_8_****/TiO_2_** **nanotube photoanode and its photocathodic protection for Q235 carbon steel under visible light**

Hong Li ^a, *^, Weizhe Song ^a^, Xingqiang Cui ^a^, Yanhui Li ^a^, Baorong Hou ^b, c^, Lianjun Cheng ^a^, Pengfei Zhang ^a^

^a^ State Key Laboratory of Bio-fibers and Eco-textiles, College of Mechanical and Electrical Engineering, Qingdao University, No. 308 Ningxia Road, Qingdao, 266071, P. R. China

^b^ Institute of Oceanology, Chinese Academy of Sciences, No. 7 Nanhai Road, Qingdao, 266071, P. R. China

^c^ Open Studio for Marine Corrosion and Protection, Pilot National Laboratory for Marine Science and Technology, No. 1 Wenhai Road, Qingdao, 266200, P. R. China

***Corresponding Author:**

*Hong Li. Email: lhqdio1987@163.com

College of Mechanical and Electrical Engineering, Qingdao University

308 Ningxia Road, Qingdao 266071, China

**Supplementary Materials：**

**Fig. S1.** SEM images of the SnIn_4_S_8_ nanosheet/TiO_2_ composite films synthesized at 180 °C for (a), (d) 3 h, (b), (e) 9 h and (c), (f) 12 h.

**Fig. S2.** PL spectra of TiO_2_ NTs and SnIn_4_S_8_/TiO_2_ composite.

**Table S1.** Comparison of previously reported catalysts for photocathodic protection.


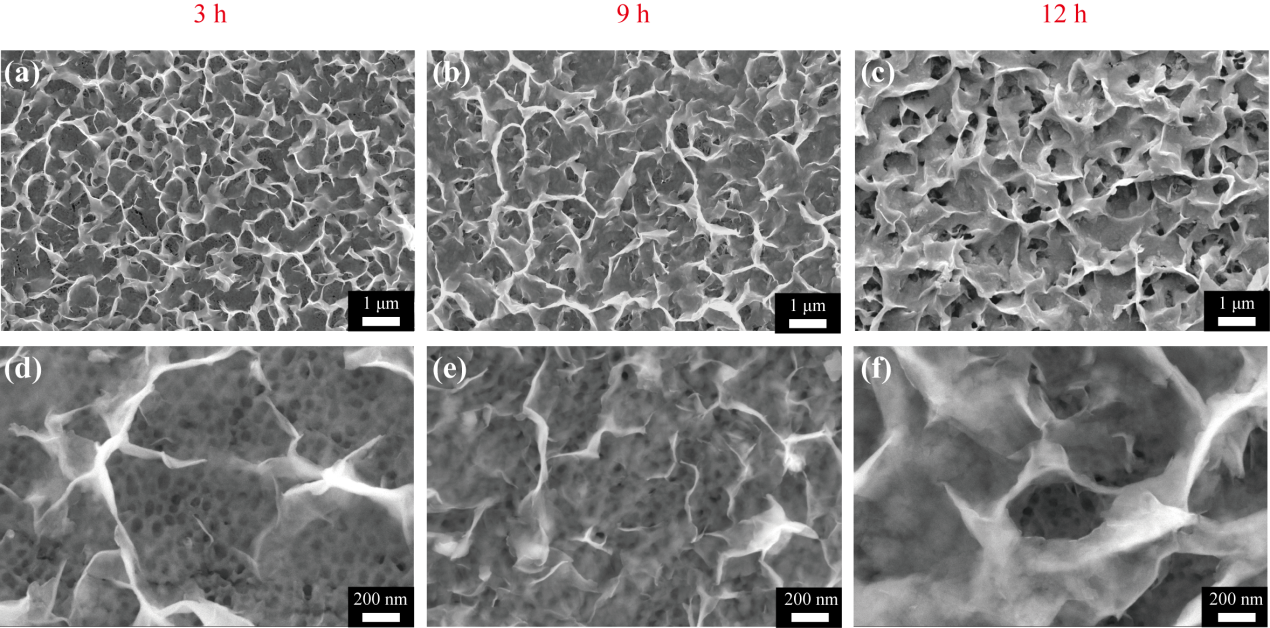


**Fig. S1.** SEM images of the SnIn_4_S_8_ nanosheet/TiO_2_ composite films synthesized at 180 °C for (a), (d) 3 h, (b), (e) 9 h and (c), (f) 12 h.


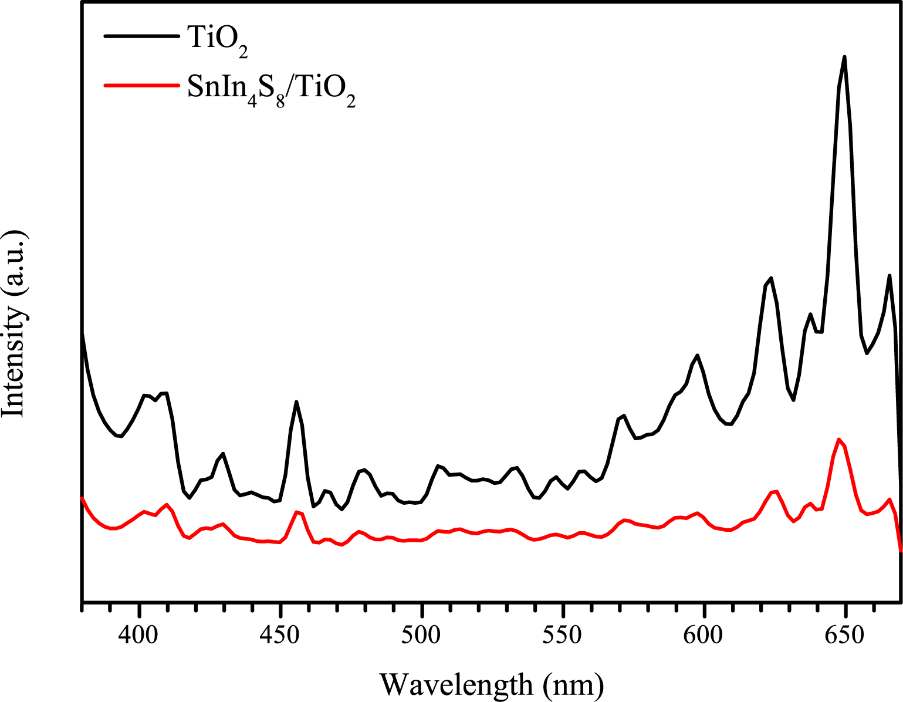


**Fig. S2.** PL spectra of TiO_2_ NTs and SnIn_4_S_8_/TiO_2_ composite.

**Table S1.** Comparison of previously reported catalysts for photocathodic protection.

| Samples | Corrosive medium | Light source | Metal | OCP drop (ΔE) | Ref |
| --- | --- | --- | --- | --- | --- |
| CdIn_2_S_4_/TiO_2_ | 3.5wt% NaCl | Xe lamp | Q235 CS | 420 mV | 1 |
| ZnIn_2_S_4_/TiO_2_ | 3.5wt% NaCl | 300W Xe lamp | Q235 CS | 360 mV | 2 |
| Ag_2_S/ZnS/ZnO | 3.5wt% NaCl | 300W Xe lamp | 304 SS | 250 mV | 3 |
| SrTiO_3_/TiO_2_ | 3.5wt% NaCl | 150W Xe lamp | 304 SS | 400 mV | 4 |
| MgTi_x_O_y_/TiO_2_ | 3.5wt% NaCl | 150W Xe lamp | 304 SS | 320 mV | 5 |
| RGO/SnO_2_/TiO_2_ | 3.5wt% NaCl | 300W Xe lamp | 304 SS | 430 mV | 6 |
| ZnS/CdTe/TiO_2_ | 0.5M NaCl | 150W Xe lamp | 403 SS | 400 mV | 7 |
| Ni-Pt/Fe-TiO_2_ | 3.5wt% NaCl | 55W Xe lamp | 403 SS | 412 mV | 8 |
| SnIn_4_S_8_/TiO_2_ | 3.5wt% NaCl | 300W Xe lamp | Q235 CS | 450 mV | This work |

**References**

[1] Z. Ma, X.M. Ma, X.T. Wang, N.Z. Liu, X.H. Liu and B.R. Hou, Study on the photocathodic protection of Q235 steel by CdIn_2_S_4_ sensitized TiO_2_ composite in splash zone, Catalysts 9 (2019) 1067−1079.

[2] H. Li, Y.H. Li, X.T. Wang and B.R. Hou, 3D ZnIn_2_S_4_ nanosheets/TiO_2_ nanotubes as photoanodes for photocathodic protection of Q235 CS with high efficiency under visible light, J. Alloy. Compd. 771 (2019) 892−899.

[3] N. Wei, Y. Lin, Z.K. Li, W.X. Sun, G.S. Zhang, M.L. Wang and H.Z. Cui, One-dimensional Ag_2_S/ZnS/ZnO nanorod array films for photocathodic protection for 304 stainless steel, J. Mater. Sci. Technol. 42 (2020) 156−162.

[4] Y.Y. Bu, Z.Y. Chen, J.P. Ao, J. Hou and M.X. Sun, Study of the photoelectrochemical cathodic protection mechanism for steel based on the SrTiO_3_-TiO_2_ composite, J. Alloy. Compd. 731 (2018) 1214−1224.

[5] C. Feng, Z.H, Chen, J.P. Jing, M.M. Sun,G.Y. Lua, J. Tian and J. Hou, A novel TiO_2_ nanotube arrays/MgTi_x_O_y_ multiphase-heterojunction film with high efficiency for photoelectrochemical cathodic protection, Corros. Sci. 166 (2020) 108441−108449.

[6] W.J. Liu, K.C. Yin, F. He, Q.X. Ru, S.X. Zuo and C. Yao, A highly efficient reduced graphene oxide/SnO_2_/TiO_2_ composite as photoanode for photocathodic protection of 304 stainless steel, Mater. Res. Bull. 113 (2019) 6−13.

[7] J. Zhang, J. Hu, Y.F. Zhu, Q. Liu, H. Zhang, R.G. Du and C.J. Lin, Fabrication of CdTe/ZnS core/shell quantum dots sensitized TiO_2_ nanotube films for photocathodic protection of stainless steel, Corros. Sci. 99 (2015) 118−124.

[8] M. M. Momeni, Y. Ghayeb and N. Moosavi, Preparation of Ni-Pt/Fe-TiO_2_ nanotube films for photoelectrochemical cathodic protection of 403 stainless steel, Nanotechnology 29 (2018) 425701−425708.
